# Enhancing Manufacturing Cell Formation Through Availability-Based Optimization Using the Black Widow Optimizer Metaheuristic

**DOI:** 10.3390/biomimetics11050294

**Published:** 2026-04-23

**Authors:** Paulo Figueroa-Torrez, Orlando Duran, Broderick Crawford, Felipe Cisternas-Caneo

**Affiliations:** 1 Departamento de Ciencias Industriales, Medio Ambiente y Energía, Universidad Católica Boliviana “San Pablo”, Colón 734, Tarija, Bolivia; 2 Escuela de Ingeniería Mecánica, Pontificia Universidad Católica de Valparaíso, Valparaíso 2362807, Chile; 3 Escuela de Ingeniería Informática, Pontificia Universidad Católica de Valparaíso, Avenida Brasil 2241, Valparaíso 2362807, Chile; broderick.crawford@pucv.cl (B.C.);

**Keywords:** manufacturing cell formation, machine availability, alternative routes, Black Widow Optimizer, multi-period optimization, metaheuristics

## Abstract

This study presents a multi-period Generalized Cell Formation Problem with Machine Availability (GCFP-MA) aimed at designing manufacturing cells that explicitly account for equipment reliability, maintainability, and temporal degradation. The proposed model extends classical formulations by introducing (i) availability-based constraints derived from Mean Time Between Failures (MTBF) and Mean Time to Repair (MTTR) and Markov-Chain models, (ii) downtime penalty costs reflecting non-production losses, and (iii) a multi-period horizon that captures system dynamics over time. To solve the resulting NP-hard problem, the Black Widow Optimizer (BWO)—a population-based metaheuristic inspired by cannibalistic reproduction—is implemented and validated against an exhaustive search benchmark. Computational experiments confirm that the BWO attains the global optimum with substantially reduced computational effort, achieving a balanced trade-off between exploration and exploitation. Results highlight that incorporating availability and repair dynamics prevents infeasible or over-optimistic configurations and yields cost-effective, robust cell layouts. The proposed approach provides both theoretical and practical contributions by integrating availability engineering and production system design within a unified optimization framework.

## 1. Introduction

Competitiveness in the manufacturing sector is complex and multifaceted, involving factors such as cost reduction and adaptation to new technologies. Innovations and more sophisticated tools and strategies are crucial to improving efficiency, which is a key determinant of competitiveness as highlighted by Gunasekaran et al. [1]. Globalization also forces companies of all sizes and sectors to compete on a global scale, requiring them to address new challenges to achieve competitiveness in line with international standards [2].

Currently, the production of goods (parts) demands high-quality standards, shorter delivery times, and greater production capacity and flexibility. Research such as Henriques and Richardson [3] indicates that proper machine distribution in the plant reduces material waste and improves workflow, directly contributing to the company’s sustainability. A well-structured distribution of machinery on the plant floor significantly enhances operational performance, thereby achieving improvements in social, environmental, and financial dimensions. Recent studies emphasize that manufacturing systems must incorporate flexibility at the design stage to cope with demand uncertainty and product variety [4]. Operations Research methods, especially mixed-integer programming and heuristics, are widely applied, though most models address static settings and focus on economic objectives [5].

A manufacturing cell is a group or cluster of machines, where the machines in each cell are selected based on production criteria, such as process compatibility, production capacity, and operational flexibility. Studies such as Durán et al. [6] highlighted that manufacturing cell formation is used to efficiently group machines on a plant floor. Organizing the machines is a complex task due to the number of combinations that can be generated. The Cell Formation Problem (CFP), as highlighted by Kusiak [7], aims to partition a plant into a predetermined number of machine cells [8]. Several studies emphasize that the cell formation problem achieves better results when integrated with scheduling, planning, and facility layout decisions, as this enables the simultaneous optimization of cell structure and production planning [9].

Das et al. [10] presented the advantages of a manufacturing cell, such as the reduction in part manufacturing times, reduction in Work-in-Process (WIP) inventories, reduction in material movements, reduction in setup times, reduction in tooling, simplification in supply management, and improvement in human relations.

Traditional CFP approaches typically assume that each part follows a single production route. However, this assumption fails to reflect the flexibility of modern manufacturing systems, where multiple machines and tools can provide alternative routes for the same part. As argued by Weckenborg et al. [4], Pinedo [11], and Safaei et al. [12], considering alternative routes increases production flexibility, optimizes resource utilization, and improves responsiveness to demand fluctuations or disruptions. Weckenborg et al. [4] show empirical or modeling evidence of how flexibility (machine routing flexibility, process flexibility, volume flexibility, etc.) improves performance in terms of throughput, responsiveness, and cost.

Consideration of these alternative routes in the CFP, according to Safaei et al. [12], extends the exploration of possible machine sequences, increasing production flexibility, optimizing resource utilization, and improving the capacity to respond to changes in demand or production interruptions.

Another critical but often neglected dimension of the CFP is machine reliability. As Ameli et al. [13] noted, machine failures can disrupt production, increase downtime, raise maintenance costs, and jeopardize delivery deadlines. Most studies on CFP design assume that machines operate without failure, resulting in unrealistic and potentially suboptimal system configurations. Shirzadi et al. [14] further emphasized that even a single failure could stop the whole process, leading to inefficiencies and higher operational costs.

In addition to routing flexibility, machine reliability constitutes a critical factor in manufacturing system performance. The increasing adoption of intelligent manufacturing cells requires highly sensorized, interconnected, and automated equipment, where reliability must be explicitly considered in clustering and selection algorithms [15,16]. Ignoring machine failures may lead to increased downtime, higher maintenance costs, and delays in meeting production deadlines [13,14].

Despite the advances made, existing models for cell formation rarely integrate both alternative routing options and machine availability in a realistic way—a measure jointly determined by the Mean Time Between Failures (MTBF) and Mean Time to Repair (MTTR). This limitation reveals a clear research gap, as current approaches cannot accurately capture the operational realities of manufacturing environments.

## 2. Literature Review

The concept of a Cellular Manufacturing System (CMS), as mentioned by Arvindh and Irani [17], is based on the segmentation of a manufacturing system into self-contained units, or cells, designed to process families of similar parts. Among the earliest approaches developed to solve the CFP is Group Technology (GT), as highlighted by Burbidge [18]. This methodology groups manufacturing elements (machines, tools, operators, etc.) into logical clusters, each dedicated to specific processing tasks (similar to CMS).

Traditionally, CFP studies assumed that each part follows a unique manufacturing route. However, this simplification overlooks the flexibility present in real systems, where machines are increasingly flexible, promoting a number of alternative ways to manufacture a part. Recognizing this, Kusiak [7] introduced the concept of multiple routing options for each part, giving rise to the Generalized Cell Formation Problem (GCFP). Their findings demonstrated that this generalization yields more coherent part families and machine groupings, particularly in large-scale problems.

The GCFP was initially formulated as an extension of the classic CFP, incorporating the flexibility characteristic of modern production systems and allowing multiple alternative routes for manufacturing the same part.

Early formal studies of the GCFP, such as Kusiak [7], proposed mathematical models that integrated multiple routes per part, thus reflecting the real complexity and flexibility of cellular manufacturing. These early formulations were integer programming models, limited in scope by the combinatorial complexity of the problem (Non-deterministic 99 Polynomial-time Hard (NP-Hard)).

During this initial stage, the GCFP solution was mainly addressed using exact methods and basic heuristics, with obvious limitations when scaling to larger and more diverse problems.

Since the beginning of this century, the application of metaheuristics has made it possible to overcome these limitations, improving the quality and scalability of solutions to the GCFP in industrial environments [19].

Currently, the most advanced studies integrate these methods with machine reliability and availability models to generate robust solutions under conditions of operational uncertainty [20].

### 2.1. Metaheuristics in the GCFP

This section focuses on studies related to the GCFP, a problem classified as NP-Hard according to Ballakur and Steudel [21], due to the large number of possible combinations that exceed the capabilities of classical optimization methods. As Shirzadi et al. [14] remarks, metaheuristics are highly recommended and widely applied to solve NP-Hard problems, including the GCFP.

One of the first metaheuristics applied to the GCFP, as shown in Lee et al. [22], was the Genetic Algorithm (GA), inspired by biological evolution and proposed by Holland [23].

Over time, new metaheuristics were introduced for GCFP. The Particle Swarm Optimization (PSO), originally proposed by Kennedy and Eberhart [24] and later applied by Kao and Chen [25], addressed the GCFP by allowing alternative process routes and by automatically identifying the number of machine cells and their grouping structures without predefinition. The PSO simulates the social behavior of bird flocks, where each particle updates its position based on inertia, its personal best, and the global best, while retaining memory of successful locations.

The Cuckoo Search (CS) algorithm was applied to the GCFP by Karoum and Elbenani [26], incorporating alternative process routings and machine reliability considerations, leading to a more realistic modeling of cellular manufacturing systems. The CS is inspired by the brood parasitism behavior of cuckoo birds. Its main strength lies in its simplicity and reduced number of parameters compared to other metaheuristics, providing an efficient mechanism for replacing inferior solutions.

In a related contribution, Jouzdani et al. [27] addressed the GCFP using Simulated Annealing (SA) by extending the classical CFP formulation to incorporate alternative process routings, machine reliability, part demands, setup costs, and detailed inter and intra-cell material handling cost structures. The SA imitates the annealing process by probabilistically accepting worse solutions, enabling the algorithm to escape local optima and avoid premature convergence.

Another approach applied to the GCFP is Tabu Search (TS), as presented by Lei and Wu [28]. In this study, the authors addressed the GCFP by considering alternative process plans within a GT framework and by incorporating system characteristics that significantly impact the optimization process. The TS, originally introduced by Glover [29], avoids revisiting recently explored regions by designating certain moves as tabu, thereby encouraging the exploration of new promising areas and permitting non-improving moves to escape local optima.

The Clonal Selection Algorithm (CSA), as explained by Karoum and Elbenani [30], was also used for the GCFP. It emulates the immune response, generating clones of good solutions, applying mutations and crossovers, and repairing unfeasible solutions, and improve it with local search to avoid getting stuck in local optima.

Finally, Figueroa-Torrez et al. [19] applied the BlackWidow Optimizer (BWO), created by Hayyolalam and Pourhaji Kazem [31], to solve the GCFP considering alternative routes and machine reliability. The BWO, inspired by the mating and cannibalism behavior of black widows, demonstrated strong performance by efficiently identifying high-quality solutions and outperforming previous metaheuristic approaches.

While metaheuristics have proven to be a powerful tool for solving the GCFP, it is equally important to take into account additional system characteristics that significantly impact production performance. Among these, availability emerges as a critical factor, as it directly affects the operational performance of the machines and the overall efficiency of the manufacturing cells. Therefore, to improve the realism and robustness of the solutions obtained through metaheuristic approaches, the incorporation of availability in the formulation of the GCFP is essential.

### 2.2. Availability in the GCFP

The availability is defined by Djenadic et al. [32] as the ability of a technical system to perform its required function under given conditions at a given time, assuming needed resources are supplied. Therefore it takes into account the probability of machine failure, as well as the time required to repair it, which provides a more realistic evaluation of performance and functionality compared to reliability alone. As stated in Figueroa-Torrez et al. [20], ignoring availability can lead to underestimating delays and associated costs, especially in production systems with sequential dependencies.

Research such as Das et al. [10] and Shirzadi et al. [14] employs an System Failure Rate (SFR) approach utilizing exponential failure distributions in GCFP. These models estimate the probability of a route’s failure by summing the failure rates of the machines involved. However, other studies related to the GCFP [26,33,34] evaluate the Breakdown Cost (BC), which quantifies failures in monetary terms using the machine’s operating time and MTBF.

Other approaches incorporate the MTTR to capture downtime related to the repair of machines belonging to a route. For example, Ameli et al. [13] and Saxena and Jain [35] apply penalties based on the MTTR, multiplying it by the failure frequency and adding the result to the total cost functions of the cells assigned to each machine (GCFP). Duplication of critical machines is another strategy proposed by Alhourani [36] and Safaei et al. [12], improving system robustness and route flexibility.

Availability becomes particularly relevant in dynamic, multiperiod models of the GCFP where machine performance degrades over time. Taking into account the MTBF and MTTR, availability-based approaches support more resilient and cost-effective cell formation decisions.

To date, only a limited number of studies have systematically integrated metaheuristics with availability metrics in the context of the GCFP. This gap represents a significant research opportunity. As highlighted in the review by Figueroa-Torrez et al. [20], the lack of integration between availability considerations and metaheuristic approaches remains evident. Incorporating availability directly into metaheuristic frameworks enables the development of more robust and practically relevant solutions to the GCFP. Such integration fosters realistic models that better capture the complexities and challenges of industrial environments. Bridging this gap holds substantial potential for enhancing decision-making in modern manufacturing systems. To this end, this study proposes a novel model of the GCFP that explicitly incorporates machine availability, referred to as Generalized Cell Formation Problem with Machine Availability (GCFP-MA). The model is evaluated through a case study using the BWO, aiming to deliver realistic and cost-effective solutions.

## 3. Mathematical Model for GCFP-MA

### 3.1. Base Model for the GCFP-MA

This study is inspired by an existing model of the Generalized Cell Formation Problem with Machine Reliability (GCFP-MR), but incorporates significant modifications. The linearized model presented by Jabal Ameli and Arkat [34], which develops a GCFP-MR with alternative routes, is adopted as the baseline model. The assumptions, parameters, decision variables, objective function and constraints are presented below.

#### 3.1.1. Assumptions

The GCFP-MR operates under the following assumptions:

- The total number of cells is defined.
- Lower and upper limits of machines per cell are known.
- Each part has at least one process route, but only one route can be selected.
- Each route consists of sequentially ordered operations performed by machines. The sequence order is crucial for determining when a part moves from one cell to another and for calculating material handling costs.
- Each part type has a defined processing time on each machine.
- Multiple identical machines are not considered.
- Production demand for each part type is deterministic and known.
- The MTBF is known for each machine.

#### 3.1.2. Mathematical Base Model

The model minimizes the total cost by considering the Intercellular Movement Cost (IMC) and BC. The IMC represents the cost generated when parts move between different manufacturing cells [37]. Specifically, it includes the cost of transferring each part between cells, as well as the cost associated with machine failures occurring along the selected production route for that part.

(a) Parameters
  The model parameters are defined as follows:
  *m*: Number of machines
  *n*: Number of parts
  *c*: Number of cells
  *r*: Total number of routes
  *$P_{i}$*: Production volume of part *i*
  $q_{i}$: Number of routes for part *i*
  $L_{l}$: Lower limit of machines in cell *l*
  $U_{l}$: Upper limit of machines in cell *l*
  $K_{ij}$: Number of machines in route *j* of part *i*
  $u_{ij}^{1}$, $u_{ij}^{2}$,…, $u_{ij}^{k_{ij}}$: Machine index in route *j* of part *i*
  $T_{ik}$: Processing time of part *i* on machine *k*
  $BC_{k}$: Machine breakdown cost *k*
  $IMC_{ij}$: Intercellular movement cost for part *i* on route *j*
  $MTBF_{k}$: Mean Time Between Failures of machine *k*.

Among the parameters, $u_{ij}^{k_{ij}}$ requires a detailed explanation for clarity. This parameter represents the set of machines *k* that are used in the production of a part *i* along a route *j*. For example, if five machines are available but only machines 1 and 5 are involved in producing part *i* on route *j*, then the corresponding values are $u_{ij}^{1}=1$ and $u_{ij}^{5}=2$, also indicating the production sequence for the part *i*.

(b) Decision Variables
  The decision variables, adapted from Jabal Ameli and Arkat [34], are defined as follows:
  $$\begin{array}{c} Z_{ij}=\left\{\begin{array}{c} 1,\;if\;route\;j\;of\;part\;i\;is\;selected\\ 0,\;otherwise \end{array}\right.\\ Y_{kl}=\left\{\begin{array}{c} 1,\;if\;machine\;k\;is\;located\;in\;cell\;l\\ 0,\;otherwise \end{array}\right.\\ X_{ijklsl}=\left\{\begin{array}{c} 1,\;if\;route\;j\;of\;part\;i\;is\;selected,\;\\ machine\;k\;is\;placed\;in\;cell\;l\;and\;\\ machine\;s\;is\;not\;in\;cell\;l\\ 0,\;otherwise \end{array}\right. \end{array}$$
(c) Objective Function
  The baseline mathematical model proposed by Jabal Ameli and Arkat [34] is defined as follows:
  *Minimize* TC =
  (1) $$\begin{array}{c} \sum_{i=1}^{n}\sum_{j=1}^{q_{i}}\sum_{k=1}^{K_{ij}-1}\sum_{l=1}^{c}\;IMC_{ij}\;P_{i}\;X_{ij\left(u_{ij}^{k_{ij}}\right)l\left(u_{ij}^{k_{ij}+1}\right)l}\;+\;\sum_{i=1}^{n}\sum_{j=1}^{q_{i}}\sum_{k=1}^{K_{ij}}\;Z_{ij}\;\frac{P_{i}\;T_{i(u_{ij}^{k_{ij}})}\;BC_{\left(u_{ij}^{k_{ij}}\right)}}{MTBF_{\left(u_{ij}^{k_{ij}}\right)}} \end{array}$$
*S.a:*
(2) $$\sum_{j=1}^{q_{i}}Z_{ij}\;=\;1\;\forall i=1,2,\ldots ,n$$
(3) $$\sum_{l=1}^{c}Y_{kl}\;=\;1\;\forall k=1,2,\ldots ,m$$
(4) $$\sum_{k=1}^{m}Y_{kl}\;\leq \;U_{l}\;\forall l=1,2,\ldots ,c$$
(5) $$\sum_{k=1}^{m}Y_{kl}\;⩾\;L_{l}\;\forall l=1,2,\ldots ,c$$
(6) $$\begin{array}{cc} X_{ijklsl}\;\leq \;Z_{ij}\;\; & \forall i=1,2,\ldots ,n;\;\forall j=1,2,\ldots ,q_{i};\;\\ & \forall k,s=1,2,\ldots ,m;\;\forall l=1,2,\ldots ,c \end{array}$$
(7) $$\begin{array}{cc} X_{ijklsl}\;\leq \;Y_{kl}\;\; & \forall i=1,2,\ldots ,n;\;\forall j=1,2,\ldots ,q_{i};\;\\ & \forall k,s=1,2,\ldots ,m;\;\forall l=1,2,\ldots ,c \end{array}$$
(8) $$\begin{array}{cc} X_{ijklsl}\;\leq \;\left(1-Y_{sl}\right)\;\; & \forall i=1,2,\ldots ,n;\;\forall j=1,2,\ldots ,q_{i};\;\\ & \forall k,s=1,2,\ldots ,m;\;\forall l=1,2,\ldots ,c \end{array}$$
(9) $$\begin{array}{cc} Z_{ij}+Y_{kl}+\left(1-Y_{sl}\right)-X_{ijklsl}\leq 2\;\; & \forall i=1,2,\ldots ,n;\;\forall j=1,2,\ldots ,q_{i};\;\\ & \forall k,s=1,2,\ldots ,m;\;\forall l=1,2,\ldots ,c \end{array}$$
(10) $$Z_{ij},Y_{kl},X_{ijklsl}\in \left\{0,1\right\}$$

The objective function, presented as Equation (1), consists of two terms. The first term represents the intercellular movement cost, while the second term accounts for the machine breakdown cost.

Constraint (2) ensures that exactly one route is selected for each part. Constraint (3) assigns each machine to exactly one manufacturing cell. Constraints (4) and (5) impose upper and lower bounds on the number of machines allowed in each cell. Constraint (6) activates the intercellular movement variables only when the corresponding route is selected. Constraint (7) links intercellular movements to the assignment of machines to cells, while Constraint (8) enforces that such movements occur only when consecutive machines belong to different cells. The constraint (9) is introduced to ensure the linearization of the model. Finally, Constraint (10) defines the binary nature of the decision variables.

### 3.2. Incorporation of Non-Production Penalty into the Objective Function

In this stage, the model is extended by incorporating a penalty for production downtime caused by machine failures. Following the approach of Saxena and Jain [35] and Ameli et al. [13], the total repair time for each route *j* of part *i* is estimated by multiplying the expected number of failures by the corresponding MTTR. This cumulative repair time is then normalized by the total production time of the part *i* ($T_{ij}$) and weighted by a penalty cost factor ($PC_{ij}$) representing the economic impact of non-production. Consequently, this new term explicitly captures the cost of unavailability within the objective function, improving the model’s ability to reflect realistic operational conditions. Accordingly, the penalty component introduced into the objective function is expressed in Equation (11).

(11) $$\sum_{i=1}^{n}\sum_{j=1}^{q_{i}}\sum_{k=1}^{K_{ij}}\;Z_{ij}\;\frac{P_{i}\;T_{i(u_{ij}^{k_{ij}})}\;MTTR_{\left(u_{ij}^{k_{ij}}\right)}\;PC_{ij}}{{MTBF}_{\left(u_{ij}^{k_{ij}}\right)}\;T_{ij}}\;$$

By incorporating Equation (11) into the objective function (Equation (1)), the updated objective function is obtained and presented in Equation (12)

*Minimize* TC =

(12) $$\begin{array}{c} \sum_{i=1}^{n}\sum_{j=1}^{q_{i}}\sum_{k=1}^{K_{ij}-1}\sum_{l=1}^{c}IMC_{ij}P_{i}X_{ij\left(u_{ij}^{k_{ij}}\right)l\left(u_{ij}^{k_{ij}+1}\right)l}+\sum_{i=1}^{n}\sum_{j=1}^{q_{i}}\sum_{k=1}^{K_{ij}}Z_{ij}\frac{P_{i}T_{i(u_{ij}^{k_{ij}})}}{{MTBF}_{\left(u_{ij}^{k_{ij}}\right)}}\left(BC_{\left(u_{ij}^{k_{ij}}\right)}+\frac{{MTTR}_{\left(u_{ij}^{k_{ij}}\right)}PC_{ij}}{T_{ij}}\right) \end{array}$$

### 3.3. Incorporation of Availability Limitation Constraints

Traditional formulations of the CFP and its generalized variants typically assume that the reliability of individual machines is sufficient to ensure overall line performance. However, this assumption neglects the interdependence among machines within a production route, where a single failure can disrupt the entire process. To overcome this limitation, the model introduces availability as a more comprehensive performance indicator that simultaneously captures the reliability and maintainability characteristics of the production system.

While previous studies have proposed reliability-based thresholds, these approaches only account for the probability of failure, disregarding the time required to restore machine functionality. As emphasized by Antosz et al. [38], availability provides a superior metric because it integrates both the failure behavior (reliability) and the repair dynamics (maintainability) of the equipment, thereby offering a more accurate measure of the system’s operational readiness. In this research, the availability of each route *j* for part *i* is calculated considering the stochastic behavior of all machines that compose that route.

**Figure 1:**
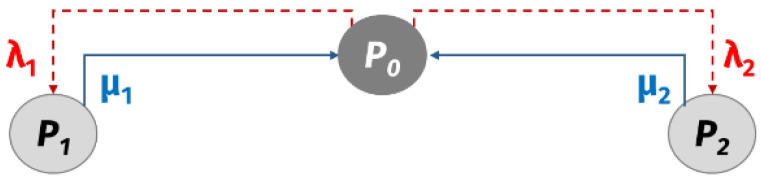
Markov chain for two machines.

To estimate the availability of a given route, we assume that both the failure rate (λ=1/MTBF) and the repair rate (μ=1/MTTR) of each machine follow an exponential distribution, as presented by Jabal Ameli et al. [39]. Under this assumption, a continuous-time Markov chain is constructed to represent the possible states of the route. Figure 1 illustrates the case of a two-machine line, where the states correspond to both machines operating ($P_{0}$), failure of machine 1 ($P_{1}$), and failure of machine 2 ($P_{2}$). The first state reflects the operational availability of the route, denoted by $RA_{ij}$.

Balancing the input and output transition rates of the Markov chain yields the steady-state probability that the route is fully operational, as defined in Equation (13):

(13) $$RA_{ij}\;=\;\frac{1}{1\;+\;\sum_{k=1}^{K_{ij}}\;\frac{\lambda_{u_{ij}^{k_{ij}}}}{\mu_{u_{ij}^{k_{ij}}}}}\;\forall i=1,2,\ldots ,n;\;\forall j=1,2,\ldots ,q_{i}$$

Equation (13) thus provides the effective availability of route *j* for part *i*, considering the combined effect of machine failures and repairs within that route.

To operationalize this concept within the optimization model, two boundary conditions are introduced. The lower availability limit (LLRA) ensures that all selected routes maintain a minimum acceptable level of operational readiness required to meet production targets, as shown in Equation (14):

(14) $$Z_{ij}\;{RA}_{ij}\;\geq LLRA\;\forall i=1,2,\ldots ,n;\;\forall j=1,2,\ldots ,q_{i}$$

Conversely, an upper availability limit (ULRA) is imposed to avoid excessive maintenance efforts and costs associated with over-maintained systems. As noted by practical maintenance studies, sustaining near-perfect availability may result in diminishing returns and unjustified cost increments as discussed by Bosse et al. [40]. Equation (15) formalizes this upper bound:

(15) $$\left(1-Z_{ij}\right)\;+\;{RA}_{ij}\;\leq ULRA\;\forall i=1,2,\ldots ,n;\;\forall j=1,2,\ldots ,q_{i}$$

Together, these two constraints enable a balanced trade-off between productivity and maintenance effort. By incorporating availability into the mathematical structure of the model, the proposed formulation provides a more realistic and economically viable representation of the manufacturing system, allowing the optimization process to select cell configurations that maximize performance while ensuring feasible and sustainable operation levels.

### 3.4. Multi-Period Objective Function

In modern manufacturing systems, logistics planning and plant distribution, as well as the definition of manufacturing cells, must be considered over the long term horizon rather than a single period. Operating conditions are constantly changing, so it is crucial to adopt a multi-period perspective. This approach allows for better anticipation of operations to maintain efficiency over time.

To implement this long-term vision, it is necessary to incorporate a multi-period approach into the mathematical model presented previously in Section 3.2. To incorporate this extension into the objective function, Equation (12) will include a summation of periods *t*, covering a total of TP periods, resulting Equation (16). Additionally, the periods *t* will be incorporated into the MTBF and MTTR calculations because machines deteriorate over time, and repair times can change.

*min TC =*

(16) $$\begin{array}{c} \sum_{t=1}^{TP}\sum_{i=1}^{n}\sum_{j=1}^{q_{i}}\sum_{k=1}^{K_{ij}-1}\sum_{l=1}^{c}IMC_{ij}P_{i}X_{ij\left(u_{ij}^{k_{ij}}\right)l\left(u_{ij}^{k_{ij}+1}\right)l}+\sum_{t=1}^{TP}\sum_{i=1}^{n}\sum_{j=1}^{q_{i}}\sum_{k=1}^{K_{ij}}Z_{ij}\frac{P_{i}T_{i(u_{ij}^{k_{ij}})}}{{MTBF}_{\left(u_{ij}^{k_{ij}}\right)}^{t}}\left(BC_{\left(u_{ij}^{k_{ij}}\right)}+\frac{{MTTR}_{\left(u_{ij}^{k_{ij}}\right)}^{t}{PC}_{ij}}{T_{ij}}\right) \end{array}$$

### 3.5. Individual Upper-Availability Limit per Route

While the incorporation of lower and upper availability limits improves the realism of the model, applying uniform thresholds across all production routes may still oversimplify the system behavior. In practice, each route *j* for part *i* can differ substantially in terms of machine composition, workload distribution, and operational complexity. Consequently, the assumption of a single global upper limit (ULRA) for all routes may lead either to over-maintained configurations or to suboptimal allocation of maintenance resources.

To overcome this limitation, the present formulation introduces individual upper-availability limits ($ULRA_{ij}$) that are specific to each route. This enhancement ensures that the model distinguishes between routes that inherently require higher availability levels—due to criticality or throughput constraints—and those for which moderate availability is sufficient to satisfy production requirements.

The rationale behind this approach is grounded in the trade-off between maintenance intensity and productive capacity. Excessive maintenance effort can unnecessarily raise costs without yielding proportional gains in production continuity, a phenomenon commonly referred to as over-maintenance. To prevent this situation, the model computes the effective demand and capacity of each route in time units and constrains the feasible solutions accordingly.

First, the total time required to meet the demand of part *i* is expressed in Equation (17):

(17) $$ULRA_{i}\;=\;\sum_{j=1}^{q_{i}}Z_{ij}\;P_{i}\;T_{ij}\;\forall i=1,2,\ldots ,n$$

Next, the model estimates the actual available capacity of each machine *k* based on its participation across multiple routes. Let $Cap_{k}$ denote the nominal capacity of machine *k* and $PoT_{ik}$ the proportion of time that machine *k* is utilized by part *i*. This proportion is defined in Equation (18):

(18) $$PoT_{ik}\;=\;\sum_{j=1}^{q_{i}}Z_{ij}\frac{P_{i}\;T_{ik}}{\sum_{i=1}^{n}\sum_{j=1}^{q_{i}}P_{i}\;T_{ik}}\;\forall i=1,2,\ldots ,n;\;\forall k=1,2,\ldots ,m$$

The available time capacity allocated to part *i* on machine *k* ($AvaCap_{ik}$) is given by Equation (19):

(19) $$AvaCap_{ik}\;=\;PoT_{ik}\;Cap_{k}\;\forall i=1,2,\ldots ,n;\;\forall k=1,2,\ldots ,m$$

Since machines degrade over time and do not always maintain their nominal capacity, the real capacity of each route *j* for part *i* is computed by adjusting the theoretical capacity with the corresponding route availability $RA_{ij}$, obtained from Equation (13). Thus, the real capacity of part *i* is obtained by Equation (20).

(20) $${RealCap}_{i}\;=\;\sum_{j=1}^{q_{i}}Z_{ij}\;{RA}_{ij}\;\sum_{k=1}^{K_{ij}}{AvaCap}_{ik}\;\forall i=1,2,\ldots ,n$$

Finally, to prevent over-maintenance, the real capacity of each route must not exceed the time-based demand requirement of part *i*. This condition defines the upper bound on availability for each route, as formulated in Equation (21):

(21) $${RealCap}_{i}\;\leq \;{ULRA}_{i}\;\forall i=1,2,\ldots ,n;\;$$

By incorporating individual upper-availability limits, the model enforces a dynamic equilibrium between production efficiency and maintenance effort. This mechanism avoids infeasible or economically unjustified configurations while ensuring that the system operates within realistic bounds of capacity utilization. As a result, the proposed formulation enhances the decision-making framework for manufacturing cell formation by promoting solutions that are not only technically feasible but also operationally sustainable and cost-effective across different production routes.

## 4. Black Widow Optimizer (BWO)

The optimization of manufacturing cell formation problems—especially when incorporating alternative routing and machine availability constraints—leads to complex, large-scale combinatorial formulations. As demonstrated by Ballakur and Steudel [21] and Shirzadi et al. [14], the GCFP belongs to the class of NP-Hard problems, where the number of feasible configurations increases exponentially with the number of machines, parts, and routes. Under such complexity, traditional exact algorithms become computationally infeasible for realistic instances, thus motivating the use of metaheuristic algorithms, which provide near-optimal solutions with reasonable computational effort.

Metaheuristics are approximate optimization methods designed to balance exploration—the global search of new promising regions—and exploitation—the refinement of already good solutions [41]. Their flexibility and problem-independent design make them particularly suitable for multi-objective and nonlinear manufacturing optimization problems, such as the GCFP-MA proposed in this study.

Among recent metaheuristics, the BWO, introduced by Hayyolalam and Pourhaji Kazem [31], has emerged as a powerful population-based algorithm inspired by the mating behavior and cannibalistic dynamics of black widow spiders. As reported by the authors, the algorithm has demonstrated competitive or superior performance compared to classical approaches such as GA, PSO, Biogeography-Based Optimization (BBO), and Artificial Bee Colony (ABC) methods, showing faster convergence and greater robustness across diverse benchmark functions.

### 4.1. Conceptual Foundations

The biological behavior underlying the BWO algorithm provides a natural mechanism for maintaining population diversity while continuously improving solution quality. During reproduction, black widows generate a large number of offspring; however, only a small subset of the strongest survive due to cannibalism—both between siblings and between parents and offspring. This survival dynamic serves as an analogy for selective pressure used in optimization, where inferior solutions are systematically eliminated to favor those with better fitness values. The process naturally balances diversification and intensification, two essential properties for escaping local optima and efficiently converging toward global solutions.

### 4.2. Algorithmic Framework

**Figure 2:**
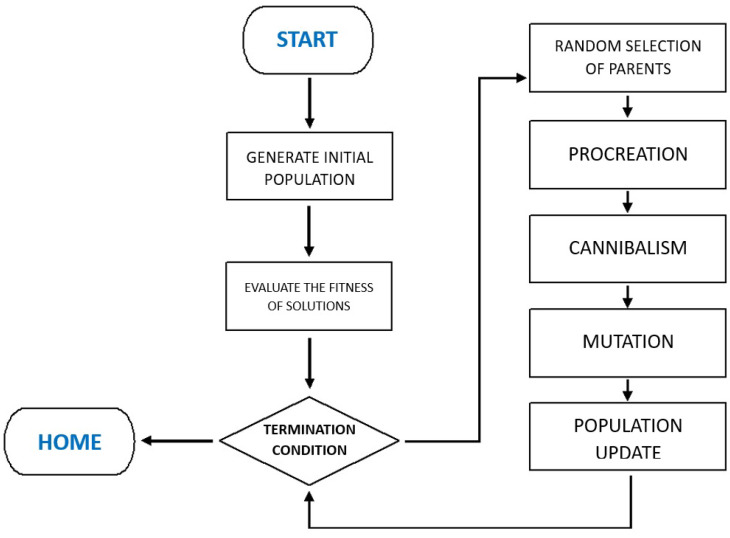
Conceptual workflow of the Black Widow Optimization (BWO) algorithm.

The BWO algorithm can be structured into five main stages: (a) initialization, (b) procreation, (c) cannibalism, (d) mutation, and (e) termination. Figure 2 illustrates the general workflow.

(a) Initialization

An initial population of candidate solutions, referred to as “widows”, is generated randomly within the feasible domain. Each widow corresponds to a potential configuration of machines, cells, and routes that satisfy the model constraints. The population matrix *W* of dimension $N_{pop}\times N_{var}$ is expressed as:

(22) $$W=\begin{array}{cc} Widows_{1} & =\\ Widows_{2} & =\\ \vdots & \\ Widows_{N_{pop}} & = \end{array}\left[\begin{array}{ccccc} w_{1,1} & w_{1,2} & w_{1,3} & \ldots & w_{1,N_{var}}\\ w_{2,1} & w_{2,2} & w_{2,3} & \ldots & w_{2,N_{var}}\\ \vdots & \vdots & \vdots & \ddots & \vdots \\ w_{N_{pop},1} & w_{N_{pop},2} & w_{N_{pop},3} & \ldots & w_{N_{pop},N_{var}} \end{array}\right]$$

where each element $w_{p,v}$ represents a decision variable, such as a route selection or a machine-cell assignment, for the *p*th candidate.

(b) Procreation

Pairs of parent solutions (${Par}_{1},{Par}_{2}$) are selected randomly and combined to generate offspring using a linear recombination mechanism controlled by a factor α∈[0,1]:

(23) $$\left\{\begin{array}{c} CH_{1}=\alpha \times {Par}_{1}+\left(1-\alpha \right)\times {Par}_{2}\\ CH_{2}=\alpha \times {Par}_{2}+\left(1-\alpha \right)\times {Par}_{1} \end{array}\right.$$

This operator encourages genetic diversity and prevents premature convergence by exploring new regions of the search space.

(c) Cannibalism

The distinctive feature of BWO lies in its cannibalistic selection. Three cannibalism mechanisms are sequentially applied:

- Sexual cannibalism, where the weaker parent is eliminated after reproduction. To facilitate understanding, the Algorithm 1 presents the corresponding pseudocode.

**Algorithm 1** Sexual Cannibalism **Input:** ${Par}_{1}$, ${Par}_{2}$ **Output:** Mother1: ${Fit}_{Par1}\leftarrow \mathrm{Calculate}\;\mathrm{Fitness}\left({Par}_{1}\right)$2: ${Fit}_{Par2}\leftarrow \mathrm{Calculate}\;\mathrm{Fitness}\left({Par}_{2}\right)$3: **if** 
${Fit}_{Par1}\leq {Fit}_{Par2}$
 **then**4:    $Mother\leftarrow Par_{1}$5: **else**6:    $Mother\leftarrow Par_{2}$7: **end if**8: **return** 
Mother

- Sibling cannibalism, where only a fraction, defined by the Cannibalism Rate (CR), of the most promising offspring survives. To facilitate understanding, the Algorithm 2 presents the corresponding pseudocode.

**Algorithm 2** Sibling Cannibalism **Input:** CH, $Number_{CH}$, CR **Output:** $Survival_{CH}$, ns1: $ns\leftarrow \mathrm{round}(CR\times Number_{CH})$▹ns must be integer2: **for** 
i=1
 **to** 
$Number_{CH}$
 **do**3:   $CH_{Fitness}\left[i\right]\leftarrow \mathrm{Calculate}\;\mathrm{Fitness}\left(CH\left[i\right]\right)$4: **end for**5: **Sort** CH▹ Based on $CH_{Fitness}$ in ascending order6: ${Survival}_{CH}\leftarrow \;\mathrm{Select}\;\mathrm{the}\;\mathrm{first}\;ns\;\mathrm{elements}\;\mathrm{from}CH$7: **return** $Survival_{CH}$, ns

- Maternal cannibalism, where superior offspring may replace the parent (mother) if their fitness is better. To facilitate understanding, the Algorithm 3 presents the corresponding pseudocode.

**Algorithm 3** Maternal Cannibalism **Input:** Mother, ${Survival}_{CH}$, ns **Output:** $Pop_{2}$1: $Pop_{2}\leftarrow Survival_{CH}$2: **for** 
i=1
 **to** 
ns
 **do**3:  ${Fitness}_{Pop2}\left[i\right]\leftarrow \mathrm{Calculate}\;\mathrm{Fitness}\left({Pop}_{2}\left[i\right]\right)$4: **end for**5: ${Fitness}_{Mother}\leftarrow \mathrm{Calculate}\;\mathrm{Fitness}\left(Mother\right)$6: **if** 
$Fitness_{Mother}<\min\left({Fitness}_{Pop2}\right)$
 **then**7:   **Insert** Mother at the beginning of ${Pop}_{2}$▹Mother becomes ${Pop}_{2}\left[0\right]$8: **end if**9: **return**
${Pop}_{2}$

These mechanisms emulate a self-regulating evolutionary pressure that accelerates convergence while maintaining diversity.

(d) Mutation

**Figure 3:**
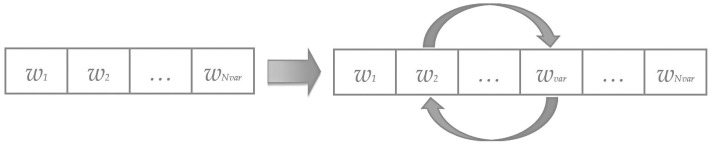
Mutation process in a *widow*.

To enhance exploration and avoid local optima, the Mutation Rate (PM) determines the fraction of individuals that undergo random perturbations. Mutation is applied by swapping two decision variables within a widow vector, generating a new configuration as it is presented in the Figure 3. This operator reintroduces variability and ensures the algorithm’s capacity to explore new feasible regions.

(e) Termination Criteria

The algorithm iteratively updates the population until one of the following stopping conditions is met:

- The maximum number of iterations is reached,
- No improvement in the best fitness value is observed for a specified number of generations, or
- A predefined optimality threshold is achieved.

The best individual identified throughout the search process is reported as the optimal or near-optimal solution. For clarity, Algorithm 4 presents the pseudocode of the BWO.

**Algorithm 4** Black Widow Optimization Algorithm  **Input:** ${Max}_{iter}$, $N_{Pop}$, *Procreating Rate* (*PR*), *Cannibalism Rate* (*CR*) and *Mutation Rate* (*PM*)  **Output:** The updated population $W^{\prime }=$$\left\{{Widow}_{1}^{\prime },{Widow}_{2}^{\prime },\ldots ,{Widow}_{N_{pop}}^{\prime }\right\}$ and Best 1: Initialization of population *W* 2: **repeat** 3:   Determine the number of reproductions (nr) based on *PR* (nr = $N_{pop}$ x PR) 4:   Select the best nr solutions in *W* and save them in ${pop}_{1}$ 5:   **for** i=1 **to** nr **do** ▹ Procreating and cannibalism 6:    Choose two *Widows* randomly as parents $\left({Par}_{1},{Par}_{2}\right)$ from ${Pop}_{1}$ 7:    Generate $N_{var}$ children using Equation (23) 8:    Eliminate the weaker father 9:    Eliminate some of the offspring based on *CR*.10:   The surviving solutions are saved in ${pop}_{2}$.11:  **end for**12:  Determine the number of mutations (nm) based on *PM* (nm = nr x PM)13:  **for** i=1 **to** nm **do** ▹ Mutation14:   Select a solution from ${pop}_{1}$15:   Randomly mutate one decision variable of the solution and generate a new solution.16:   Mutated solutions are saved in ${pop}_{3}$17:  **end for**18:  Update $W={pop}_{2}+{pop}_{3}$19: **until** a termination criterion is met20: **return** the best Widow from *W*

### 4.3. Advantages and Suitability for the GCFP-MA

The BWO algorithm’s combination of aggressive selection pressure and controlled diversification makes it particularly well suited for the GCFP-MA. Its simple structure, the small number of control parameters, and its ability to avoid getting stuck in local optima enable efficient handling of highly constrained, nonlinear optimization problems.

Moreover, its population-based nature allows the simultaneous exploration of multiple machine-cell configurations, facilitating the identification of solutions that balance manufacturing cost, availability, and intercellular movement.

In the next section, the algorithm is adapted and implemented specifically for the proposed multi-period GCFP-MA model, followed by an extensive computational experiment to evaluate its performance in terms of solution quality, convergence behavior, and computational efficiency.

## 5. Experimental Phase

### 5.1. Case Study Data

To evaluate the performance of the proposed multi-period GCFP-MA model, a numerical case study was developed. The initial dataset was adapted from Ameli et al. [13] and Karoum and Elbenani [30], incorporating the operational characteristics of nine machines and eight parts, as presented in Table 1. The dataset includes the MTBF, MTTR and breakdown costs for each machine, as shown in Table 2. These values serve as baseline inputs for the reliability and maintainability components of the model.

To better reflect real-world manufacturing conditions, the study introduces a temporal dimension comprising five discrete planning periods. The failure and repair rates of each machine vary across periods, simulating progressive wear, maintenance effects, and learning curves in repair operations. The Table 3, Table 4, Table 5 and Table 6 are introduced to present the corresponding MTBF and MTTR values that are updated accordingly for each period to represent degradation and recovery dynamics over time. Additionally, the nominal capacity of each machine ($Cap_{k}$) was considered, as reported in Table 7.

This temporal modeling enables the evaluation of the GCFP-MA under evolving operational conditions, thus enhancing the model’s practical relevance.

Additionally, the lower and upper route availability limits were specified to constrain feasible solutions. The lower limit (LLRA) was set to 0.75 for the case study, ensuring that all production routes maintain a minimum acceptable level of operational readiness. This parameter is externally defined and typically established by decision-makers according to company-specific policies related to availability, service level, or quality requirements. Therefore, it is treated as an input parameter rather than a decision variable within the model. The individual upper limits ($ULRA_{ij}$) were computed using Equation (17), and are presented in Table 8, based on the time-based production requirements for each part and route. These upper limits prevent over-maintenance scenarios where excessive availability leads to unnecessary cost increases, thus ensuring a feasible level of production capacity.

The penalty costs ($PC_{ij}$) were defined as proportional to the number of machines participating in each route and scaled by a factor of five to represent the cost impact of production interruptions. For this case study, this scaling was adopted to provide a consistent representation of downtime costs; however, it is not a universal value and may vary depending on the specific cost structure and operational context of each application. This configuration aligns with prior works [13,35] and ensures that both production and downtime costs are realistically captured in the model. The $PC_{ij}$ for each route are presented in Table 9.

The complete dataset described in the previous section is used as input for three metaheuristic algorithms, which are implemented and compared using Python 3.11.8 in Visual Studio Code 1.114.0.

### 5.2. Metaheuristic Parameter Settings

In addition to the BWO, which was introduced in Section 4, two additional metaheuristics were considered for comparison purposes: GA and Linear Population Size Reduction Success-History based Adaptive Differential Evolution (L-SHADE).

The GA was chosen because of its frequent use and proven effectiveness in solving the CFP and its variants [42,43]. Its evolutionary structure and ability to explore complex combinatorial search spaces have made it one of the most commonly used metaheuristics in this domain. Parent selection was performed using a rank-based roulette wheel selection mechanism. The crossover operator was implemented as a single-point crossover, where two parent solutions exchange genetic material at a randomly selected crossover point. Mutation was applied with a probability defined at the gene level.

On the other hand, L-SHADE was selected due to its strong performance in continuous and high-dimensional optimization problems. As an advanced variant of Differential Evolution (DE), L-SHADE incorporates adaptive parameter control based on historical success and a dynamic reduction of population size, which enhances convergence speed and solution quality. Although it has not been applied to CFP, L-SHADE has demonstrated state-of-the-art performance in continuous optimization, ranking first in the CEC 2014 competition [44] on real-parameter single-objective optimization [45,46].

For all metaheuristics, the parameter settings were defined based on configurations reported in the literature. Considering that the case presented in the previous section has a dimension of D=17 (8 parts and 9 machines), the parameters are shown in Table 10, Table 11 and Table 12.

The parameter settings were defined based on configurations reported in the literature: Hazarika and Laha [47] for GA, Tanabe and Fukunaga [48] for L-SHADE, and Figueroa-Torrez et al. [19] for BWO. All three metaheuristics were evaluated against the known optimal solution of 28,984.81, which was obtained through an Exhaustive Search (ES) procedure evaluating 474,600 possible solutions.

### 5.3. Results and Statistical Analysis

Due to the stochastic nature of metaheuristic algorithms, the performance of a single run may not be representative of the overall behavior of the method. Therefore, 100 independent runs were carried out, each initialized with different random populations, allowing the assessment of both solution quality and robustness. This experimental design was adopted to avoid biased results and to ensure a statistically reliable comparison among the evaluated metaheuristics.

For each run, the best objective function value obtained at the final iteration was recorded. These values were then used to compute statistical performance indicators, including the mean, Standard Deviation (STD), Mean Absolute Error (MAE), Root Mean Square Error (RMSE), and Relative Percentage Deviation (RPD) [49], which measures the deviation of the average solution quality with respect to the known optimal solution (28,984.81) and is presented in Equation (24).

(24) $$RPD=\frac{{\bar{Z}}_{Metaheuristic}-Z_{opt}}{Z_{opt}}\times 100$$

#### 5.3.1. Performance Indicators

The statistical results are presented in Table 13.

Table 13 summarizes the statistical performance of the evaluated metaheuristics over 100 independent runs. Significant differences can be observed in terms of solution quality, variability, and robustness.

The GA exhibits the weakest performance among the evaluated methods. It presents the highest mean value (29,250.766), as well as the largest dispersion (STD = 385.613), confirming its limited capability compared with the other metaheuristics. Additionally, its worst-case performance (30,640.400) is more distant from the optimal solution compared with the others.

In contrast, both L-SHADE and the BWO demonstrate strong performance, achieving mean values close to the optimal solution. Among them, BWO obtains the best average performance (28,965.380), followed closely by L-SHADE (28,982.178). Furthermore, BWO shows the lowest variability (STD = 100.691), suggesting a more stable behavior.

This trend is further supported by the error-based metrics. The BWO achieves the lowest values of MAE (40.571) and RMSE (108.089), indicating a smaller deviation from the optimal solution across runs. Similarly, the RPD confirms this behavior, with BWO presenting the lowest average deviation (0.140%), followed by L-SHADE (0.198%) and GA (1.127%).

#### 5.3.2. Analysis of Optimal Solution Attainment

In addition to the statistical performance indicators, the reliability of each metaheuristic was evaluated by measuring the percentage of runs that successfully reached the known optimal solution. This metric (hit rate) reflects the consistency and robustness of each algorithm under stochastic conditions.

Table 14 presents the proportion of successful runs for each metaheuristic. The results indicate significant differences in performance among the evaluated methods.

The GA exhibits a notably low hit rate of only 10%, indicating a limited ability to consistently identify the global optimum. In contrast, L-SHADE achieved the highest hit rate (82%), demonstrating strong convergence reliability. The BWO also showed a high hit rate (72%), confirming its robustness, although slightly lower than L-SHADE.

**Figure 4:**
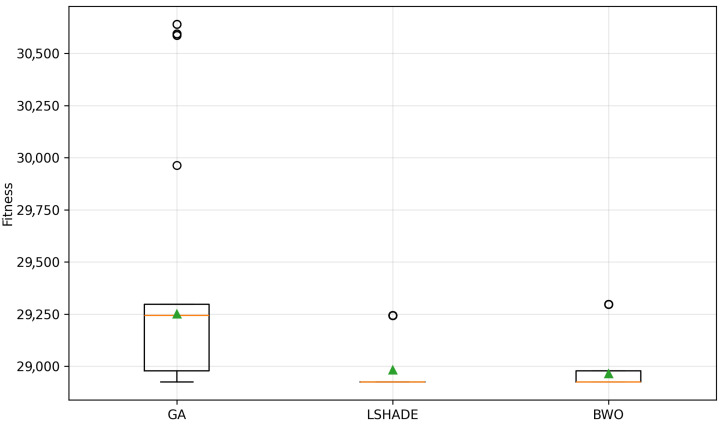
Fitness distribution per metaheuristic.

To provide a visual representation of these results, a boxplot of the fitness distribution for each metaheuristic is presented in Figure 4, where the circles indicate outliers and green triangles represent the mean values.

Figure 4 shows that the GA exhibits a wide interquartile range and several extreme outliers, indicating high variability and inconsistent performance. In contrast, both L-SHADE and BWO present more compact distributions.

The L-SHADE shows the most concentrated distribution around lower fitness values, confirming its stability and accuracy. Although BWO also demonstrates strong performance, its distribution is slightly more dispersed compared to L-SHADE. These observations are consistent with the statistical indicators presented in Table 13.

#### 5.3.3. Time Analysis

In addition to solution quality, the computational efficiency of each metaheuristic was evaluated by analyzing the execution time required to complete each run. Table 15 presents the best, mean, STD, and worst execution times obtained over 100 independent runs.

The results reveal substantial differences in computational cost among the evaluated algorithms. The L-SHADE exhibits significantly higher execution times due to its large population size ($N_{Pop}=306$) and high number of function evaluations ($NFE_{max}=$ 170,000).

In contrast, both GA and BWO demonstrate considerably lower execution times. The BWO achieves the best computational performance, with a mean time of 0.575 s and low variability (STD = 0.102), indicating fast and stable execution.

**Figure 5:**
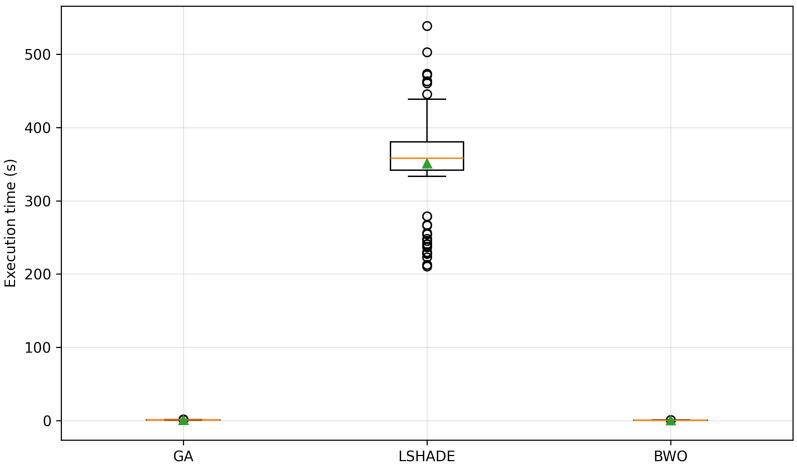
Execution time distribution per metaheuristic.

To provide a visual comparison, the distribution of execution times is illustrated in Figure 5.

Figure 5 shows that L-SHADE presents a wide dispersion and multiple outliers, indicating high computational cost and variability. In contrast, GA and BWO exhibit highly concentrated distributions.

Overall, the results highlight a clear trade-off between solution quality and computational cost. While L-SHADE demonstrates strong convergence reliability, this performance is achieved at a significantly higher computational expense.

On the other hand, the BWO provides a highly competitive balance between accuracy and efficiency, achieving near-optimal solutions with minimal computational resources. Therefore, from a cost-effectiveness perspective, BWO emerges as the most suitable approach for practical applications.

## 6. Conclusions and Future Work

This study developed and validated a multi-period GCFP-MA, solved using the BWO. The proposed framework advances traditional cell formation methodologies by integrating three key elements often neglected in prior literature:

- The explicit modeling of machine availability as a function of reliability and maintainability.
- The incorporation of downtime penalties that quantify the economic cost of non-production.
- The adoption of a multi-period perspective that captures the temporal evolution of machine performance and system costs.

The model was tested on a benchmark case derived from the literature, demonstrating the feasibility and robustness of the proposed formulation. The BWO algorithm efficiently converged to the global optimum, previously verified through ES, confirming its effectiveness in addressing high-dimensional optimization problems. The integration of availability-based constraints and temporal deterioration dynamics yielded solutions that more accurately reflect real manufacturing conditions, avoiding unrealistic or excessively optimistic scenarios that are often generated by static models.

To further assess the performance of the proposed approach, a comparative analysis was conducted against two well-established metaheuristics: the GA and L-SHADE. The results demonstrated that while L-SHADE achieved the highest reliability in reaching the optimal solution, the BWO consistently provided the best balance between solution quality and computational efficiency.

From a solution quality perspective, BWO obtained the lowest average deviation from the optimal solution, as reflected by its superior values in RPD, mean objective function, and error-based metrics such as MAE and RMSE. Additionally, it exhibited the lowest variability across runs, indicating a stable and robust search behavior.

From a computational standpoint, BWO significantly outperformed L-SHADE, requiring substantially lower execution times while maintaining comparable or superior solution quality. Although GA showed low computational cost, its performance was notably inferior in terms of accuracy and consistency, limiting its applicability for this type of problem.

These findings highlight the importance of evaluating both solution quality and computational cost when selecting optimization methods. While advanced algorithms such as L-SHADE offer strong convergence capabilities, their high computational demand may restrict their practical use. In contrast, BWO emerges as a cost-effective alternative, delivering high-quality solutions with minimal computational resources.

From a theoretical perspective, this research contributes to the literature by introducing availability as a central decision dimension in cellular manufacturing system design. It bridges the gap between availability engineering and operations research, providing a unified modeling framework that simultaneously considers cost, performance, and system resilience.

From a practical standpoint, the GCFP-MA offers decision-makers a quantitative tool to anticipate the long-term effects of machine degradation and maintenance strategies on production system configuration. The results emphasize the relevance of incorporating dynamic availability into the design of flexible manufacturing systems, particularly in environments where machine reliability plays a critical role in operational continuity.

Finally, the metaheuristic framework presented in this study expands the set of available tools for solving complex manufacturing optimization problems. The BWO, through its balance of exploration and exploitation mechanisms, proved to be efficient, stable, and adaptable, making it a promising approach for addressing real-world industrial applications.

### Future Research Directions

Several research directions naturally emerge from this work:

1. *Dynamic Demand and Uncertainty:* Future studies could incorporate stochastic or time-varying demand to evaluate how uncertainty affects the robustness of cell configurations and route selections. This would allow assessing the performance of the proposed model under more realistic and volatile production environments.
2. *Integration with Maintenance Scheduling:* Coupling the GCFP-MA with preventive or predictive maintenance models would enable the joint optimization of production planning and maintenance policies, strengthening the link between reliability management and production efficiency.
3. *Multi-Objective Optimization:* Extending the model toward a multi-objective framework—including sustainability, energy consumption, or carbon footprint—would align with current industrial and Environmental (ESG) priorities.
4. *Comparative Metaheuristic Analysis:* While the BWO demonstrated strong performance, future research could expand the comparative analysis to include other state-of-the-art metaheuristics and hybrid approaches (e.g., Golden Eagle Optimizer (GEO) or hybrid swarm-based methods), enabling a deeper evaluation of convergence speed, robustness, and solution diversity.
5. *Endogenous Parameter Estimation:* The current model assumes that certain parameters are externally provided, such as the LLRA and the PC associated with unrealized production. Future work could focus on developing estimation models or analytical formulations to determine these parameters based on historical data, operational conditions, or reliability models.
6. *Advanced Failure and Repair Modeling:* The current formulation relies on exponential distributions to model machine failures and repairs. However, real manufacturing systems often exhibit non-memoryless behavior, which can be better captured using distributions such as Weibull or lognormal. Incorporating these distributions would improve the realism and applicability of the model.
7. *Industrial Implementation:* Applying the proposed model to real manufacturing environments—such as automotive or mining component production—would provide empirical validation of its practical value. Additionally, integration with digital twin technologies could enable real-time monitoring and adaptive optimization.

## Figures and Tables

**Table 1:** Sequence and processing time dataset based on Ameli et al. [13].

Sequence/ Processing Time (min)	Parts (Volume)																			
	P1(75)			P2 (130)			P3 (110)		P4 (145)		P5 (110)		P6 (105)		P7 (140)				P8 (115)	
Machines	R1	R2	R3	R1	R2	R3	R1	R2	R1	R2	R1	R2	R1	R2	R1	R2	R3	R4	R1	R2
**M1**	1(5)			1(4)			1(4)	1(4)	1(5)	1(5)		1(4)		1(4)	1(5)			1(5)	1(4)	
**M2**		1(5)	1(5)		1(5)	1(5)					1(3)		1(5)			1(3)	1(3)	2(3)		1(4)
**M3**							2(3)	2(3)	2(3)	2(3)										
**M4**	2(3)			2(4)				3(3)	3(5)	3(5)		2(4)			2(4)		2(4)			
**M5**	3(4)		2(4)	3(3)	2(3)	2(3)	3(4)			4(4)					3(3)		3(3)			
**M6**		2(5)														2(4)		3(4)	2(3)	2(4)
**M7**											2(5)	3(5)	2(5)	2(5)						
**M8**		3(4)		4(4)		3(4)	4(3)	4(3)	4(3)	5(4)			3(5)	3(5)			4(5)			
**M9**	4(5)		3(5)		3(3)						3(5)	4(5)			4(5)	3(5)		4(5)		
**Intercellular** **cost (** IMC **)**	**375**	**375**	**0**	**1300**	**0**	**650**	**1100**	**0**	**0**	**1450**	**1100**	**550**	**525**	**0**	**700**	**0**	**700**	**700**	**575**	**0**

**Table 2:** MTBF, BC and MTTR dataset based on Ameli et al. [13].

Machine	MTBF ^1^ (h)	BC ^2^	MTTR ^3^ (h)
M1	90	900	3
M2	51	2000	8
M3	73	2000	6
M4	60	1600	7
M5	76	1500	2
M6	62	1800	4
M7	71	1400	5
M8	58	1700	3
M9	65	1500	5

^1^ Mean time between failure; ^2^ Breakdown cost.; ^3^ Mean time to repair.

**Table 3:** Failure rate for five periods.

	Period 1	Period 2	Period 3	Period 4	Period 5
**Machine 1**	0.01111	0.01111	0.01111	0.01111	0.01111
**Machine 2**	0.01961	0.01923	0.01887	0.01852	0.01818
**Machine 3**	0.01370	0.01408	0.01449	0.01493	0.01538
**Machine 4**	0.01667	0.01695	0.01724	0.01754	0.01786
**Machine 5**	0.01316	0.01316	0.01316	0.01316	0.01316
**Machine 6**	0.01613	0.01613	0.01613	0.01613	0.01613
**Machine 7**	0.01408	0.01429	0.01449	0.01471	0.01493
**Machine 8**	0.01724	0.01695	0.01667	0.01639	0.01613
**Machine 9**	0.01538	0.01538	0.01538	0.01538	0.01538

**Table 4:** Repair rate for five periods.

	Period 1	Period 2	Period 3	Period 4	Period 5
**Machine 1**	0.33333	0.33333	0.33333	0.33333	0.33333
**Machine 2**	0.12500	0.12500	0.14286	0.14286	0.14286
**Machine 3**	0.16667	0.16667	0.14286	0.14286	0.14286
**Machine 4**	0.14286	0.14286	0.12500	0.12500	0.12500
**Machine 5**	0.50000	0.50000	0.50000	0.50000	0.50000
**Machine 6**	0.25000	0.25000	0.25000	0.25000	0.25000
**Machine 7**	0.20000	0.20000	0.16667	0.16667	0.16667
**Machine 8**	0.33333	0.33333	0.50000	0.50000	0.50000
**Machine 9**	0.20000	0.20000	0.20000	0.20000	0.20000

**Table 5:** MTBF for five periods.

	Period 1	Period 2	Period 3	Period 4	Period 5
**Machine 1**	90	90	90	90	90
**Machine 2**	51	52	53	54	55
**Machine 3**	73	71	69	67	65
**Machine 4**	60	59	58	57	56
**Machine 5**	76	76	76	76	76
**Machine 6**	62	62	62	62	62
**Machine 7**	71	70	69	68	67
**Machine 8**	58	59	60	61	62
**Machine 9**	65	65	65	65	65

**Table 6:** MTTR for five periods.

	Period 1	Period 2	Period 3	Period 4	Period 5
**Machine 1**	3	3	3	3	3
**Machine 2**	8	8	7	7	7
**Machine 3**	6	6	7	7	7
**Machine 4**	7	7	8	8	8
**Machine 5**	2	2	2	2	2
**Machine 6**	4	4	4	4	4
**Machine 7**	5	5	6	6	6
**Machine 8**	3	3	2	2	2
**Machine 9**	5	5	5	5	5

**Table 7:** Machine capacities.

M1	M2	M3	M4	M5	M6	M7	M8	M9
1300	800	700	1400	1300	300	1000	800	1100

**Table 8:** $ULRA_{ij}$ for each route *j* from part *i*.

P1			P2			P3		P4		P5		P6		P7				P8	
R1	R2	R3	R1	R2	R3	R1	R2	R1	R2	R1	R2	R1	R2	R1	R2	R3	R4	R1	R2
1275	1050	1050	1950	1430	1560	1540	1430	2320	3045	1430	1980	1575	1470	2380	1680	2100	2380	805	920

**Table 9:** Production cost of each part *i* on each route *j*.

P1			P2			P3		P4		P5		P6		P7				P8	
R1	R2	R3	R1	R2	R3	R1	R2	R1	R2	R1	R2	R1	R2	R1	R2	R3	R4	R1	R2
20	15	15	20	15	15	20	20	20	25	15	20	15	15	20	15	20	20	10	10

**Table 10:** GA parameter settings used in the experimental phase.

Parameter	Calculation	Value
Population size ($N_{Pop}$)	–	20
Maximum number of iterations ($N_{iter}$)	$N_{iter}=2\times n_{Parts}\times m_{Machines}$	144
Probability of mutation ($P_{M\text{-}gen}$)	–	0.015

**Table 11:** L-SHADE parameter settings used in the experimental phase.

Parameter	Calculation	Value
External archive size factor ($r_{arc}$)	–	2.6
Initial population size factor ($r_{N_{\mathrm{init}}}$)	–	18
Initial population size ($N_{Pop,init}$)	$N_{Pop,init}=D\times r_{N_{\mathrm{init}}}$	306
Minimum population size ($N_{Pop,min}$)	–	4
*p*-best selection rate (*p*)	–	0.11
Maximum number of function evaluations ($NFE_{\max}$)	$NFE_{\max}=D\times 10,000$	170,000
Standard deviation of crossover rate distribution ($\sigma_{CR}$)	–	0.1
Standard deviation of mutation factor distribution ($\sigma_{F}$)	–	0.1
Size of historical memory (*H*)	–	6

**Table 12:** BWO parameter settings used in the experimental phase.

Parameter	Calculation	Value
Population size ($N_{Pop}$)	–	100
Number of iterations ($N_{iter}$)	–	75
Procreation rate (PR)	–	0.8
Cannibalism rate (CR)	–	0.4
Mutation rate (PM)	–	0.4
Number of procreations (nr)	$nr=N_{Pop}\times PR$	80
Number of surviving offspring per reproduction (ns)	ns=round(CR×D)	7
Number of mutations (nm)	nm=nr×PM	32

**Table 13:** Performance indicators obtained from 100 independent runs.

	GA	L-SHADE	BWO
Best	28,924.809	28,924.809	28,924.810
Worst	30,640.400	29,243.528	29,297.360
Mean	29,250.766	28,982.178	28,965.380
STD	385.613	123.065	100.691
MAE	325.957	57.370	40.571
RMSE	503.446	135.221	108.089
RPD	1.127	0.198	0.140

**Table 14:** Percentage of runs reaching the optimal solution.

Metaheuristic	Hit Rate (%)
GA	10
L-SHADE	82
BWO	72

**Table 15:** Computational time (in seconds) obtained from 100 independent runs.

	GA	L-SHADE	BWO
Best time (s)	0.686	210.188	0.459
Worst time (s)	1.642	538.665	0.934
Mean time (s)	0.987	350.796	0.575
STD time (s)	0.183	64.764	0.102

## Data Availability

Data available on request from the authors. The data that support the findings of this study are available from the corresponding author, O.D., upon reasonable request.

## Abbreviations

**Acronym**	**Definition**
ABC	Artificial Bee Colony
BBO	Biogeography Based Optimization
BC	Breakdown Cost
BWO	Black Widow Optimizer
CFP	Cell Formation Problem
CMS	Cellular Manufacturing System
CR	Cannibalism Rate
CS	Cuckoo Search
CSA	Clonal Selection Algorithm
DE	Differential Evolution
ES	Exhaustive Search
ESG	Environmental, Social and Governance
FF	Firefly Optimization
GA	Genetic Algorithm
GCFP	Generalized Cell Formation Problem
GCFP-MA	Generalized Cell Formation Problem with Machine Availability
GCFP-MR	Generalized Cell Formation Problem with Machine Reliability
GEO	Golden Eagle Optimizer
GT	Group Technology
IMC	Intercellular Movement Cost
L-SHADE	Linear Population Size Reduction Success-History based Adaptive Differential Evolution
MAE	Mean Absolute Error
MTBF	Mean Time Between Failures
MTTR	Mean Time to Repair
PM	Mutation Rate
NP-Hard	Non-deterministic Polynomial-time Hard
PSO	Particle Swarm Optimization
RMSE	Root Mean Square Error
RPD	Relative Percentage Deviation
SA	Simulated Annealing
SFR	System Failure Rate
STD	Standard Deviation
TS	Tabu Search
WIP	Work-in-process
